# Spine Stereotactic Radiosurgery for Metastatic Pheochromocytoma

**DOI:** 10.7759/cureus.4742

**Published:** 2019-05-23

**Authors:** Shane Mesko, Brian J Deegan, Neil M D'Souza, Amol J Ghia, Bhavana V Chapman, Behrang Amini, Mary Frances McAleer, Xin A Wang, Paul D Brown, Claudio E Tatsui, Laurence Rhines, Jing Li

**Affiliations:** 1 Radiation Oncology, The University of Texas MD Anderson Cancer Center, Houston, USA; 2 Radiation Oncology, Mays Cancer Center, University of Texas, San Antonio, USA; 3 Radiology, The University of Texas MD Anderson Cancer Center, Houston, USA; 4 Radiation Physics, The University of Texas MD Anderson Cancer Center, Houston, USA; 5 Radiation Oncology, Mayo Clinic, Rochester, USA; 6 Neurosurgery, The University of Texas MD Anderson Cancer Center, Houston, USA

**Keywords:** ssrs, stereotactic radiation, pheochromocytoma, spine stereotactic radiosurgery, metastatic pheochromocytoma

## Abstract

Purpose: Despite aggressive primary treatment, up to 13.5% of patients diagnosed with pheochromocytoma may develop metastases, most often affecting the axial skeleton. Given that systemic therapy options are often inadequate, local therapy remains the cornerstone of palliation for these patients. Historically poor responses to standard fractionated radiotherapy have led to the consideration of stereotactic radiosurgery as an option to overcome potential radioresistance and provide durable local control of these tumors. Here we report our institutional experience in treating spine metastases from pheochromocytoma with spine stereotactic radiosurgery (SSRS).

Methods and materials: Our clinical databases were retrospectively reviewed for patients with metastatic pheochromocytoma treated with SSRS from 2000-2017. Seven patients with 16 treated metastatic spinal lesions were identified. Local control was evaluated using magnetic resonance imaging (MRI). Pain and symptom data were assessed to evaluate toxicity using Common Terminology Criteria for Adverse Events (CTCAE) v4.03. The Kaplan-Meier method was used to assess local control and overall survival (OS).

Results: Median follow-up for treated lesions was 11 months (range 2.2 - 70.8). Most lesions were treated to a dose of 27 Gy in three fractions (62.5%). Other fractionation schemes included 24 Gy in one fraction (25%), 16 Gy in one fraction (6.3%), and 18 Gy in three fractions (6.3%). Treatment sites included the cervical spine (18.8%), thoracic spine (37.5%), lumbar spine (31.3%), and sacrum (12.5%). The crude local control rate was 93.7%, with one thoracic spine lesion progressing 20.7 months after treatment with 24 Gy in one fraction. Kaplan-Meier OS rates at 1 and 2 years after SSRS were 71.4% and 42.9%, respectively. Most common toxicities included acute grade 1-2 pain and fatigue. There was one case of vertebral fracture in a cervical spine lesion treated to 27 Gy in three fractions, which was managed non-surgically.

Conclusion: Very few studies have explored the use of SSRS in metastatic pheochromocytoma. Our data suggest this modern radiation modality is effective, safe, and provides durable local control to palliate symptoms and potentially limit further metastatic seeding. Larger patient numbers and longer follow-up will further define the role of SSRS as a treatment option in these patients.

## Introduction

Pheochromocytoma is a catecholamine-secreting tumor originating from chromaffin cells in the adrenal medulla and belongs to the intra-adrenal subcategory of paragangliomas (PGLs) [1-2]. The classic presenting triad is episodic headache, sweating, and palpitations in the context of persistent hypertension [3]. Pheochromocytomas are rare with only 1-6 cases per million people per year, and approximately 13.5% of these are found to be metastatic [3-5].

Five-year survival for metastatic disease ranges from 34% to 60%, depending largely on organ involvement, compared to 90% to 95% survival rates for benign disease [6]. Bone metastases carry the most favorable prognosis while visceral metastases portend comparatively poor survival [7-9]. Surgery remains the mainstay of therapy for both benign and malignant disease. However, recurrence rates for metastatic disease are high even after resection, and the goals of therapy are often geared toward palliation of symptoms [10]. Iodine^131^-labeled metaiodobenzylguanidine (I^131^-MIBG) has also shown utility as an adjunct to surgery [9,11]. Systemic therapy, including chemotherapy with cyclophosphamide, vincristine, and dacarbazine (CVD), has shown short-lived benefit, while targeted therapies directed against vascular endothelial growth factor (VEGF) and platelet-derived growth factor receptors (PDGF-R) are under investigation [2,12].

The role of radiotherapy in pheochromocytoma is poorly defined. Although external beam radiation is commonly utilized in head and neck parasympathetic PGLs, evidence for benefit in pheochromocytoma is poor. A 1978 review on early reports of external radiotherapy in metastatic pheochromocytoma concluded that pheochromocytoma is most likely a radiation-resistant tumor, but that radiotherapy may be beneficial for symptom control in bone and lymph node metastasis when delivered at high doses [13]. Due to the rarity of malignant pheochromocytoma, studies with sizable cohorts are difficult to assemble and are consequently scarce, particularly in the radiation literature. Radiotherapy advances, especially with respect to image guidance, intensity modulated radiation therapy (IMRT), and spine stereotactic radiosurgery (SSRS), could prove valuable in the treatment of malignant pheochromocytoma [14-15]. SSRS, in particular, is an attractive option to overcome traditional radioresistance and provide more durable local control of metastatic pheochromocytoma. The purpose of this series is to report our institutional experience using SSRS to treat spine metastases from pheochromocytoma in an effort to provide evidence of the viability and benefit of this option in these rare cases.

## Materials and methods

Study design and patients

The available records of patients with histologically confirmed metastatic pheochromocytoma treated with SSRS between 2000 and 2017 were retrospectively reviewed in an institutional review board (IRB) approved analysis. 

Data sources

Patient demographics, treatment date(s), location, and dose prescription information were sourced from institutional databases. Histological confirmation, oncologic history, clinical surveillance, toxicity outcomes, and survival data were obtained via review of the medical records.

Treatment

Patients were treated using IMRT with multi-modality image guidance. The details of patient setup, treatment planning, and delivery have been previously described [15-16]. In short, patients were immobilized using a full body vacuum cradle (BlueBAG, Elekta, Stockholm, Sweden). In addition, a thermoplastic mask (High Precision System for Head, Neck and Shoulders, Orfit Industries America, Wijnegem, Belgium) or plastic body cover sheet (BodyFIX, Elekta, Stockholm, Sweden) was added for precise patient positioning and immobilization. Spine magnetic resonance imaging (MRI) was used for target and critical structure delineation. Images were fused to simulation computed tomography (CT) scan for dose calculation and IMRT planning. The IMRT plan was optimized in Pinnacle treatment planning software (Version 9.8 or 9.10, Philips Radiation Oncology Systems, Andover, MA) using 7 to 9 posterior beams with step-and-shoot technique. Multi-modality image guidance was achieved with either CT-on-rails, Varian on-board imaging system (Varian Medical Systems, Palo Alto, CA), and/or Brainlab ExacTrac targeting system (Brainlab AG, Feldkirchen, Germany). Alignment was assessed by the treating radiation oncologist. Stereoscopic imaging (ExacTrac) was used to monitor and adjust intra-fractional patient motion. A dedicated radiation physicist was also present to oversee the work flow and perform the alignment.

Treatment was prescribed to the gross tumor volume (GTV) which was defined as all radiographically evident tumor including bony, paraspinal soft tissue, and epidural disease as based on image-fusion data sets. The clinical treatment volume (CTV) included the GTV plus an additional expansion to include adjacent contiguous bone marrow at risk per the International Spine Radiosurgery Consortium guidelines [17]. The GTVs were prescribed to receive either 18 Gy or 27 Gy in three fractions under the multifraction protocol, while the single fraction treatments consisted of either 24 Gy or 18 Gy. SSRS was not given as a boost after conventional radiation in any case. Spinal cord and cauda equina were contoured as true structures rather than thecal sac and no planning target volume (PTV) was used. Dose constraints for normal structures per our institutional standard depending on fractionation and prior treatment [14]. Multi-fraction treatments were delivered on consecutive or alternating days depending on machine availability and patient convenience.

Outcomes and assessment

Follow-up for the cohort consisted of MRI and clinical exam every three months for the first two years, then every 3-6 months thereafter. Patients could have had additional surveillance imaging performed at the discretion of the treating physician. End points included local control, overall survival, and toxicity. Local recurrence was evaluated with spine MRI, interpreted by the reading radiologist, and confirmed by the radiation oncologist in the context of the original SSRS treatment plan. Pain and symptom data were assessed to evaluate toxicity and were scored using the Common Terminology Criteria for Adverse Events (CTCAE) version 4.03. Toxicity was recorded if radiation therapy was a possible cause, but excluded if attributed to disease progression. Local control and overall survival (OS) were analyzed using the Kaplan-Meier method.

## Results

Patient and treatment characteristics

Baseline patient characteristics are listed in Table 1. Ultimately, seven patients with a total of 16 treated lesions were deemed suitable for retrospective analysis. The median age at the time of SSRS was 58 years (range: 46 - 68 years). All patients were of good performance status at the time of radiation treatment with a median Karnofsky Performance Status (KPS) of 90. Two patients presented with metastatic disease at the time of initial diagnosis, while the other five had a median time to first metastasis of 62.9 months (range 19.3-178) (Table 2). All patients in the series had undergone upfront surgical resection of their primary disease and had received systemic chemotherapy and exhibited systemically progressive disease at the time of SSRS.

**Table 1:** Patient characteristics KPS: Karnofsky Performance Status

Patient	Age	Sex	KPS	Lesions treated
1	63	Male	80	1
2	46	Male	90	5
3	60	Male	100	4
4	50	Male	80	1
5	56	Female	90	2
6	68	Male	90	2
7	58	Male	90	1

**Table 2:** Patient survival and toxicity SSRS: Spine stereotactic radiosurgery

Patient	Time to SSRS from Metastasis (months)	Vital Status	Total Survival from Metastasis (months)	Survival from SSRS (months)	Toxicity (grade)
1	25.4	Deceased	36.4	11.0	Pain (2), Fatigue (1)
2	9.1	Deceased	85.8	76.7	None
3	72.7	Deceased	86.1	13.4	C5 Fracture (2), Pain (2), Fatigue (1)
4	9.8	Deceased	39.4	29.6	None
5	13.3	Deceased	16.5	3.2	None
6	19.5	Deceased	43.3	23.8	None
7	6.8	Alive	48.2	41.1	Pain (2)

The most common SSRS fractionation scheme was 27 Gy in three fractions (62.5%). Other fractionation schemes included 24 Gy in one fraction (25%), 16 Gy in one fraction (6.3%) and 18 Gy in three fractions (6.3%). Treatment sites included the cervical-spine (18.8%), thoracic-spine (37.5%), lumbar-spine (31.3%) and sacrum (12.5%) (Table 3). The median number of vertebral levels treated per course was two (range 1-3). 

**Table 3:** Spine stereotactic radiosurgery (SSRS) treatment regimens, sites, and local control

Patient	Treatment Area	Dose and Fractionation	Local Control	Freedom from Failure (months)
1	C5-C7	27 Gy (9 Gy x 3)	Yes	6.4
2	L2	16 Gy (16 Gy x 1)	Yes	65.1
	L1, L3	27 Gy (9 Gy x 3)	Yes	25.3
	T8-T10	24 Gy (24 Gy x 1)	No	20.7
	T1	24 Gy (24 Gy x 1)	Yes	3.6
	T11-T12	27 Gy (9 Gy x 3)	Yes	3.6
3	C5	27 Gy (9 Gy x 3)	Yes	11.0
	T7	27 Gy (9 Gy x 3)	Yes	11.0
	T5	27 Gy (9 Gy x 3)	Yes	10.7
	S1-S2	18 Gy (6 Gy x 3)	Yes	10.7
4	L3	24 Gy (24 Gy x 1)	Yes	25.4
5	L4-T5	27 Gy (9 Gy x 3)	Yes	2.3
	T3-T5	27 Gy (9 Gy x 3)	Yes	2.2
6	S3-S5	27 Gy (9 Gy x 3)	Yes	20.7
	C1	27 Gy (9 Gy x 3)	Yes	16.8
7	L3-L4	24 Gy (24 Gy x 1)	Yes	37.6

Tumor control

Median follow-up for each treated site was 11 months (range: 2 - 71 months) (Table 3). Crude local control rate was 93.7% with one treatment failure observed during the follow-up period. This solitary local failure occurred 21 months post-treatment in a patient treated with 24 Gy in one fraction. Six of the seven patients treated died during the course of follow-up. and no deaths were considered treatment related. Kaplan-Meier OS at one and two years after SSRS were 71.4% and 42.9%, respectively.

Toxicity

Toxicity in this cohort was minimal and categorically low grade (CTCAE grade 2 or lower; Table 2). There were three cases of grade 2 non-radicular, musculoskeletal-type pain. Two patients experienced grade 1 fatigue after treatment and one patient developed a grade 2 vertebral compression fracture at the level of C5, which was managed non-operatively. Overall, treatment was well tolerated with little associated morbidity.

## Discussion

The traditional treatment modalities for metastatic pheochromocytoma consist of surgical resection or debulking in conjunction with I^131^-MIBG. Combinations of CVD triplet chemotherapy are also added in some cases [18]. The role of external beam radiation therapy (EBRT) has been scarcely examined and remains a subject of debate. PGLs as a whole are typically considered slowly proliferating tumors and as such, the efficacy of radiotherapy, in comparison to surgery, is best assessed by radiographic evolution and symptomatic responses rather than the degree of tumor regression [19]. While recognizing the role of radiotherapy in PGLs of the head and neck is well established - a review of more than 1000 tumors reported near 90% local control rate at 10-year follow with a total dose of 40 to 45 Gy - the role of radiation in non-head and neck PGLs is less clear, particularly in the metastatic setting [20]. An influential 1978 review by Drasin examined case reports describing EBRT in metastatic pheochromocytoma, finding the evidence to be lacking in support of EBRT for treatment of the primary tumor, but more favorable for palliative treatment of bony metastases [13]. Although the Drasin review suggested that higher doses may be more efficacious, high-dose EBRT utilizing older technology has been associated with considerable toxicity, especially in the retroperitoneum [21]. Various case reports have since described the effectiveness of modern high-dose radiotherapy in the treatment of metastatic pheochromocytoma to the urinary bladder, liver, lymph nodes, and lumbar spine [22-25]. A recent review of 17 patients with metastases of pheochromocytoma in the thorax, abdomen, or pelvis who were treated with a variety of modern radiotherapy techniques, including IMRT, showed EBRT to be effective for local control and symptomatic relief in 76% of patients especially in conjunction with I^131-^MIBG in cases of bulky tumors [26].

In this study, we describe the promising role of a modern modality in radiation therapy, stereotactic radiosurgery (SRS), in the management of metastatic pheochromocytoma to the axial skeleton. A local control rate of 93.5% was achieved for these treated segments using a variety of fractionation schemes. In the recent past, SRS has been successfully applied to the management of jugular PGLs, contributing to disease stabilization and even regression [19,27-28]. In contrast, the use of SSRS in the treatment of malignant pheochromocytoma is a relatively unexplored entity. A recent retrospective review by Vogel et al. in 2014 compiled together 24 patients with malignant pheochromocytoma and PGL who were treated with various forms of EBRT [6]. Of these patients, only one was treated with SSRS to bony metastases, specifically to C3 at a dose of 24 Gy, and two others received SRS for metastases to the liver and the organ of Zuckerkandl. As a group, these three patients treated with SRS achieved more durable local control in comparison to patients receiving standard fractionation. Our results corroborate these findings and further point the effectiveness in terms of local control of SRSS in treatment of spine metastases in particular. With respect to the lone treatment failure seen in our sample, this occurred in the context of florid systemic progression 21 months after treatment, and may represent re-seeding rather than SSRS failure.

Toxicities in our study were limited to grade 2. Three out of six patients experienced some form of treatment-related toxicity, with both pain and fatigue (grade 2 and grade 1, respectively) occurring most frequently. All three patients who received SSRS within 1 year of the development of metastasis survived beyond two years post-treatment. These results suggest there may be a benefit to early therapeutic intervention with SSRS in both local control and overall disease progression.

Limitations of this study include the relatively small number of cases which effectively precludes extensive statistical analysis. These limitations are unsurprising given the rarity of this condition and the limited utilization of radiotherapy in the overall management of pheochromocytoma. Future studies may better define the long-term toxicities of treatment, the durability of local control, and optimal timing/context for integration of radiation therapy.

## Conclusions

To our knowledge, this is one of the first studies describing the utility of SSRS in the treatment of metastatic pheochromocytoma. Our data suggest that SSRS is an effective, safe, and durable treatment option. Despite strong local control, pheochromocytoma remains a progressive, metastatic disease requiring improvements in systemic therapy. Given the robust tumor control of SSRS and the possibility for metastatic foci to serve as sources for further systemic spread, proactive treatment of spinal metastasis earlier in the disease course may offer therapeutic benefit to these patients. Larger patient numbers and longer follow-up is required to address this issue in the future.
